# Deep learning using inductively coupled plasma spectroscopy spectra accurately predicts various soil physicochemical properties for soil diagnosis

**DOI:** 10.1038/s41598-025-24274-3

**Published:** 2025-11-20

**Authors:** Satoshi Nakamura, Akihiro Imaya, Kenta Ikazaki

**Affiliations:** 1 Japan International Research Center for Agricultural Sciences (JIRCAS), 1-1 Owashi, Tsukuba, Ibaraki 305-8686 Japan; 2 Forest Research and Management Organization , Forestry and Forest Products Research Institute (FFPRI-FRMO), 1 Matsunosato, Tsukuba, Ibaraki 305-8687 Japan

**Keywords:** Soil diagnosis, Tropics, Inductively coupled plasma spectroscopy, Deep learning, Environmental chemistry, Agroecology, Environmental monitoring

## Abstract

Improving soil diagnosis-based agriculture can help reduce fertilizer utilization and its environmental impact. However, conventional soil diagnostic methods are time-consuming and expensive, which limits their application. Although various rapid soil testing methods have been suggested, their accuracy remains largely unexplored. Herein, multiple soil parameters were predicted using the spectral data obtained from inductively coupled plasma (ICP) spectroscopy combined with deep learning. We analyzed 1941 soil samples from seven countries with various land-use patterns and histories. All ICP wavelength spectral data from the 1 M NH_4_OAc extract were used for deep learning. The targeted soil properties included exchangeable bases (Ca, Mg, K, and Na); pH (H_2_O); pH (KCl); electrical conductivity; available P (Bray1-P); exchangeable Al; cation exchange capacity; total carbon, nitrogen, clay, and sand contents. The predicted soil parameters were consistent with the observations. Most soil parameters had determination coefficients (*R*^2^) of > 0.9, and the lowest *R*^2^ (0.81; total carbon) was relatively high. To our knowledge, this is the first study to demonstrate the prediction of multiple soil parameters using the ICP spectra of soil extracts. Our accurate predictions indicate that this method can be applied for precise, affordable, and rapid soil diagnosis, which could enhance soil-diagnosis-based agriculture.

**Supplementary Information:**

The online version contains supplementary material available at 10.1038/s41598-025-24274-3.

## Introduction

Soil diagnosis is essential for improving crop productivity. Soil nutrient conditions must be monitored to enhance crop production and ensure appropriate fertilizer application, for example, by addressing excessive, insufficient, or incorrect fertilizer application^1^. In addition, it is essential to understand the specific soil parameters in each field for precision and smart agricultural settings, which are becoming increasingly widespread globally^2^. Therefore, the demand for soil diagnosis and the prediction of soil parameters is expected to increase. However, soil diagnosis has not been used comprehensively to date because official soil testing methods are complex and relatively expensive per sample^1^. This can be attributed to the diverse soil parameters applied in soil diagnosis and the different associated extraction solutions and determination procedures for each parameter. For example, exchangeable cations are generally extracted using 1 M ammonium acetate (NH_4_OAc) at pH 7, whereas exchangeable Al is extracted using 1 M potassium chloride (KCl) solution. The elemental concentrations of these extracts must also be quantified using various apparatus. In addition, it often requires considerable time and effort to re-dilute and re-analyze the extracts owing to the limitations associated with detectable concentration ranges. Such factors considerably increase labor and time costs. The cost requirements of soil analysis hamper soil diagnosis-based agriculture, especially in developing countries.

Numerous trials have been conducted to determine multiple soil properties using spectral data, such as near-infrared (NIR)^3–5^, visible/near-infrared (VNIR)^6^, and mid-infrared spectra (MIR)^7^, in addition to diffuse reflectance spectroscopy^8^ and laser-induced fluorescence spectrum^9^. Such studies have demonstrated that reflectance spectroscopy techniques can facilitate the development of technologies for instantaneous and non-destructive measurement of soil parameters and, in turn, facilitate remote sensing using satellite sensors or unmanned aerial vehicles. However, to date, predictions made using reflectance spectra have only been applied to certain soil parameters. For example, Yang et al.^6^ suggested that VNIR spectrum prediction has a very good or good predictive capacity for soil organic matter, clay, sand, silt, total nitrogen (N), available N, and total potassium (K); however, it is not appropriate for cation exchange capacity (CEC), available phosphorus (P), or soil pH.

The wet extraction method for soil analysis is more complex than reflectance spectroscopy and requires a large time and cost due to various extractions. Inductively coupled plasma atomic emission spectroscopy (ICP-AES) is a multi-purpose elemental analysis method capable of simultaneously measuring multiple elements in a liquid sample. It has been used to determine cations and other elements such as Al and Fe in soil analyses. During ICP-AES, concentrations are determined by designating the measurement wavelength of the target element. However, such a simultaneous approach acquires and stocks all wavelength data. Currently, these potentially invaluable data are discarded because they do not provide direct information on soil chemical properties. Recently, studies have demonstrated that plasma emission spectra combined with machine learning can be used for material identification in other fields, such as the differentiation of biological tissues using cold atmospheric plasma emission^10^. These examples highlight the potential of full-spectrum plasma data for diagnostic purposes, although such approaches have not yet been applied to comprehensive soil diagnosis.

This study aims to predict multiple parameters of soil physicochemical properties using all-wavelength spectral data obtained from ICP-AES. The target analysis parameters were soil pH (H_2_O and KCl extraction), soil electrical conductivity (EC), available P (Bray I method), total carbon (C), total N, exchangeable bases (Ca, Mg, Na, and K), exchangeable Al, CEC, and particle size distribution (clay and sand fractions). We compared the prediction accuracy of our method with that of reflectance spectroscopy methods, particularly MIR spectroscopy, which has a relatively high accuracy for multiple parameter predictions^11^.

## Materials and methods

### Soil samples

We used 1941 soil samples from tropical and subtropical environments collected by the Japan International Research Center for Agricultural Sciences (JIRCAS) from 2016 to 2020. The number of soil samples from each country was as follows: Burkina Faso (1,073 samples), Laos (334 samples), Japan (298 samples), Mozambique (115 samples), Palau (59 samples), Madagascar (49 samples), and the Philippines (13 samples) (Suppl. Table 1). Samples were collected from agricultural and forest soils for various purposes, such as soil classification and evaluation of technology implementation. Therefore, the sample set included surface and profile soil samples and covered various fertilizer application histories. The number of samples used for the analysis of each parameter (Suppl. Table 1) differed because of the different demands of each activity.

In this study, we used 80% of the samples as training data and the remaining 20% as evaluation data. We split the data using completely random sampling. The variance indicators for the training and evaluation data are listed in Table 1.

**Table 1:** Sample variation in randomly selected sample-sets for training and evaluation.

Parameter		Count			Average		Median		Std		CV		Max		Min	
		Tr	Ev	Total	Tr	Ev	Tr	Ev	Tr	Ev	Tr	Ev	Tr	Ev	Tr	Ev
pH(H_2_O)		1534	407	1941	6.00	6.04	5.88	5.88	0.80	0.81	0.13	0.13	8.53	8.51	4.09	4.34
pH(KCl)		1534	407	1941	4.82	4.81	4.62	4.54	0.87	0.87	0.18	0.18	7.65	7.60	3.46	3.43
EC	mS m^−1^	1534	407	1941	16.47	16.02	2.52	2.64	31.88	31.29	1.94	1.95	228.50	183.60	0.19	0.54
Bray 1P	mg kg^−1^	1535	407	1942	10.99	11.62	2.30	2.60	28.82	29.45	2.62	2.54	257.73	268.28	0.00	0.07
T-C	%	1474	341	1815	1.031	0.968	0.647	0.628	1.195	0.871	1.160	0.900	27.912	5.913	0.013	0.054
T-N	%	1474	341	1815	0.099	0.094	0.072	0.066	0.105	0.074	1.059	0.785	2.522	0.407	0.005	0.007
Ex-Ca	cmolc kg^−1^	1535	407	1942	7.14	7.34	2.68	3.06	13.85	12.43	1.94	1.69	290.99	88.11	0.01	0.01
Ex-K	cmolc kg^−1^	1535	407	1942	0.81	0.80	0.18	0.18	2.12	2.09	2.62	2.60	19.26	16.81	0.00	0.00
Ex-Mg	cmolc kg^−1^	1535	407	1942	1.58	1.64	0.70	0.80	2.56	2.58	1.62	1.57	20.02	20.02	0.00	0.00
Ex-Na	cmolc kg^−1^	1535	407	1942	0.42	0.40	0.06	0.06	1.16	1.00	2.75	2.50	15.15	7.68	0.00	0.00
Ex-Al	cmolc kg^−1^	1535	407	1942	0.68	0.72	0.23	0.24	1.27	1.36	1.86	1.88	8.28	7.83	0.00	0.00
CEC	cmolc kg^−1^	1535	407	1942	12.08	12.60	9.93	10.28	9.78	10.16	0.81	0.81	90.81	58.40	0.36	0.31
Sand	%	683	175	858	50.00	49.64	50.92	50.40	24.28	24.68	0.49	0.50	96.03	93.96	3.56	3.22
Clay	%	679	173	852	31.18	30.28	28.37	27.12	18.44	18.32	0.59	0.61	90.35	85.74	1.98	4.23

Tr: samples used for training, Ev: samples used for evaluation.

### Method of soil chemical analysis

The soil samples were air-dried for approximately 2 weeks. Coarse organic debris and gravel were removed from the air-dried soil, after which they were sieved through a 2-mm diameter sieve. Subsequently, a series of soil physicochemical analyses were performed. Briefly, soil pH was measured at a soil-to-solution ratio of 1:2.5, using distilled water for pH (H_2_O) and 1 M KCl for pH (KCl), using a glass electrode pH meter (LAQUA F-72, HORIBA, Ltd., Kyoto, Japan). Total C and N contents were determined using the dry combustion method with an NC analyzer (Sumigraph NC-220, Sumika Chemical Analysis Service, Ltd., Japan). Available P was extracted according to the Bray-1 method^12^ and determined using the ascorbic acid-molybdenum blue method with a spectrophotometer (UV-1900; Shimadzu Corp., Kyoto, Japan). Exchangeable Al was extracted with a 1 M KCl solution and determined using inductively coupled plasma atomic emission spectroscopy (ICP-AES) (ICPE-9000, Shimadzu Corp.)^13^. Exchangeable bases were extracted using a 1.0 M NH_4_OAc solution (pH 7). The cation concentrations were determined using ICP-AES (ICPE-9000, Shimadzu Corp.). CEC was determined using the following procedure. After exchangeable base extraction, the residues were washed with 80% methanol. Subsequently, saturated NH_4_^+^ was extracted four times with 10% KCl (pH 7.0), and the CEC was determined using the salicylate method^14^, through continuous flow analysis using an Auto Analyzer III (BL-TEC K. K., Japan). Particle size distributions and clay and sand fractions were analyzed using wet sieving and the pipette method. Although coarse and fine sands were determined separately, the data were merged into the total sand fraction.

### Data collection procedure of ICP spectra

**Fig. 1:**
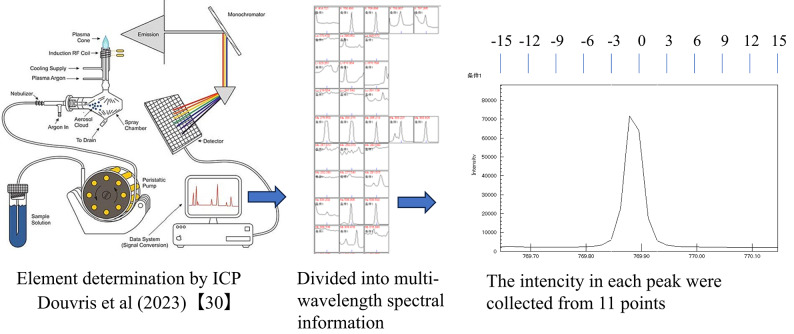
Image of all wavelength data collection from the ICP.

The spectral intensity data were collected at all wavelengths via exchangeable base analysis extracted by 1 M NH_4_OAc solution, using inductively coupled plasma atomic emission spectroscopy (ICPE Solutions v. 2.11; Shimadzu Corp.). The measurement conditions for ICP-AES were as follows: radio frequency (RF) power of 1.2 kW, axial observation position, introduction rate of plasma gas of 10.0 L/min, and carrier gas of 0.70 L/min, using a pneumatic nebulizer. This enabled the intensities of 232 wavelengths and 71 target elements to be determined by default. Two wavelengths with sensitivity information for C shared by Shimadzu Corp. were added, resulting in 234 wavelength targets. The peak profile data for 31 pixels centered at each measurement wavelength were obtained for each determination. In this trial, individual intensity values were obtained at 11 pixels with relative positions from the center pixel of 0, 3, 6, 9, 12, 15, − 3, − 6, − 9, − 12, and − 15 by specifying the integration range of the peak intensity calculation to be one pixel for each peak profile data (Fig. 1). These 11 pixels were selected because they were considered sufficient to preserve the peak shape while reducing computational load. The natural logarithms (log_*e*_* x*) of each of the obtained intensity values were calculated and used as explanatory variables (x; 2574 pixels/sample). An intensity value of 1,000,000 was assumed for luminescence intensity saturation. The intensity value was set to 1 for samples with negative intensity values. The actual measured values of each analysis item were used as dependent variables (y). The maximum and minimum correlation coefficients between the natural log-transformed intensity values and each wavelength are shown in Supplementary Table 2.

### Machine learning procedure

**Fig. 2:**
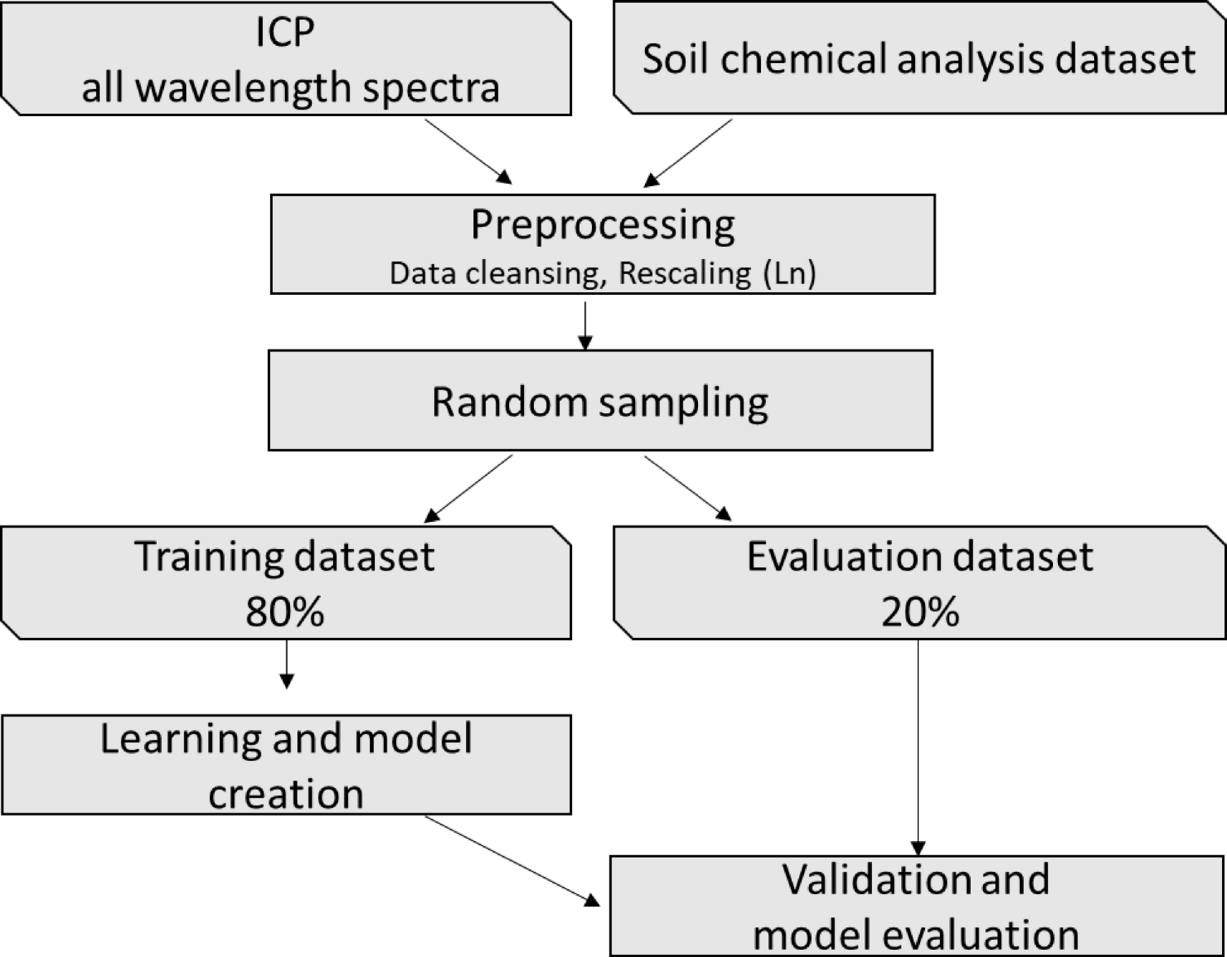
Overview of the model construction method.

The feedforward neural network (FFNN) approach was used to predict the soil properties using Neural Network Console v1.7.7352.44102 (NNC; Sony Network Communications Inc., Japan). An outline of the model establishment procedure is shown in Fig. 2. “Adam”, an algorithm for first-order gradient-based optimization, was used as the optimization algorithm for the FFNN^15^ with a learning rate of 0.001. A rectified linear unit (ReLU) was used as the activation function^16^, with dropout (*p* = 0.2) to avoid overfitting^17^. The Huber loss function^18^, which is a loss function for robust estimation, was used. The initial FFNN structure constructed by the authors consisted of four hidden layers with ReLU activation in addition to the input layer, and the output layer employed Huber loss. In the final model selected through automatic optimization using Neural Network Console, the hidden layers contained 10,000, 5,000, 1,000, 100, and 20 neurons, respectively, followed by an output layer with a single neuron. The network architecture was optimized for each soil property prediction through a random search–based hyperparameter optimization, with a maximum of 1000 epochs and a batch size of 64.

A randomly selected training dataset (80% of the entire sample) of the actual values and spectral data was learned using the NNC. After learning the training dataset, all the properties were predicted using the spectrum of the evaluation samples (20% of the entire sample). The accuracy of the predicted properties was evaluated by comparing them with the observed properties obtained using the procedure described below.

### Model evaluation metrics

The accuracies of the predicted parameters were evaluated using several indices such as the determination coefficient (*R*^2^), root mean squared error (RMSE), and mean absolute error (MAE). The residual predictive deviation (RPD) and range error ratio (RER) were calculated according to Malley et al.^19^ and Chang et al.^20^ to evaluate the applicability of the model for soil diagnosis. The indices used to evaluate the accuracy of the prediction methods were calculated using the following equations:

1 $${R}^{2}=1-{\sum }_{i=1}^{n}{\left({y}_{m}-{y}_{p}\right)}^{2}/{\sum }_{i=1}^{n}{\left({y}_{m}-\overline{{y }_{p}}\right)}^{2}$$

2 $$RMSE=\sqrt{\frac{1}{n}{\sum }_{i=1}^{n}{\left({y}_{m}-{y}_{p}\right)}^{2}}$$

3 $$MAE=\frac{1}{n}\sum_{i=1}^{n}\left|{y}_{m}-{y}_{p}\right|$$

where *Y*_m_ is a given soil property measured using conventional soil analysis, and *Y*_*p*_ is the soil property predicted by deep learning using ICP spectrum data.

4 $$RPD=S.D./RMSE$$

5 $$RER=Range/RMSE$$

where S.D. is the standard deviation of the sample in question.

## Results and discussion

### Prediction accuracy for each soil parameter

#### Exchangeable bases

**Fig. 3:**
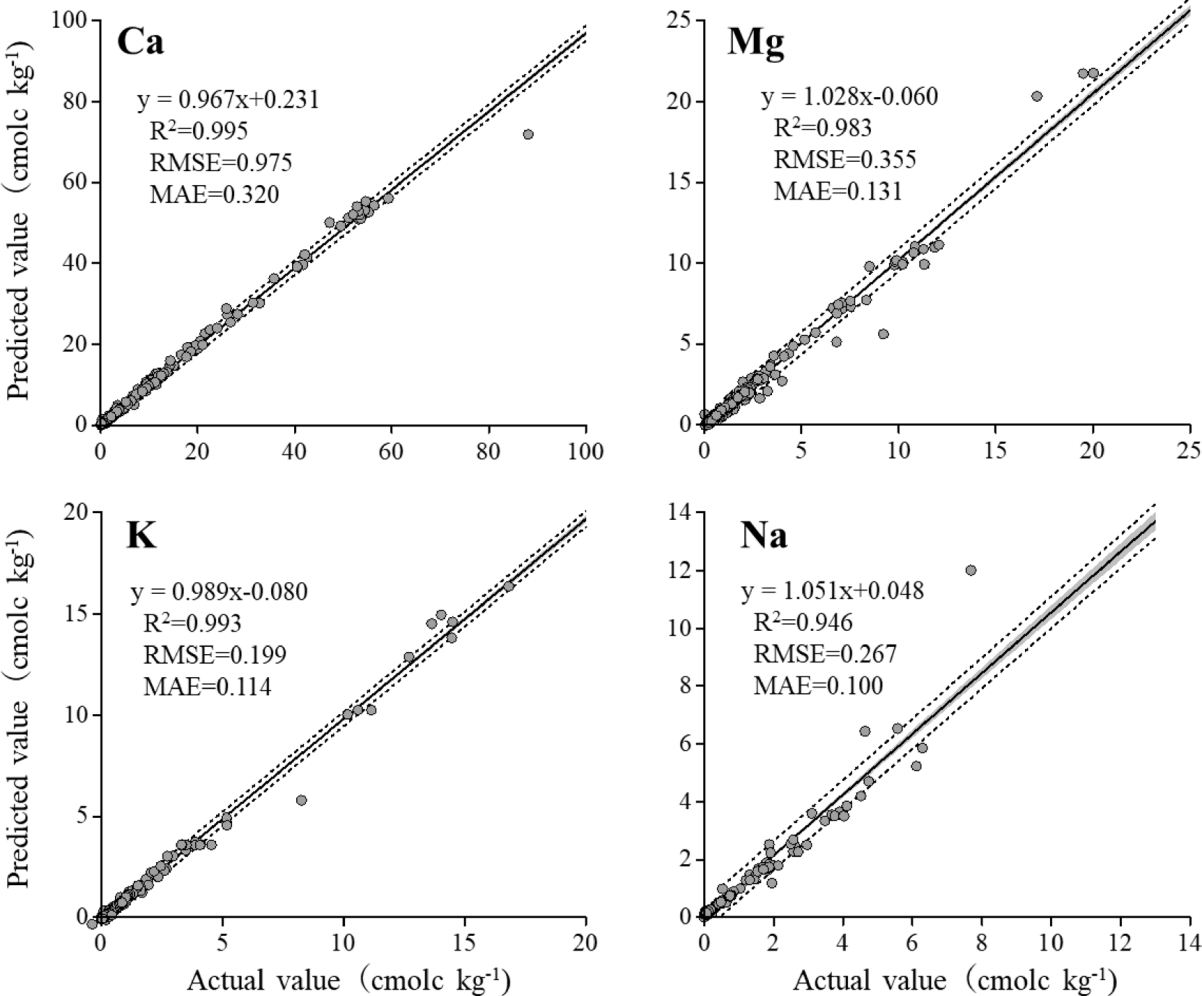
Scatter plots of laboratory-measured values versus ICP spectrum base predicted values of exchangeable cations. Grey zones indicate 95% confidence interval, dotted lines indicate 95% prediction interval.

The exchangeable cations predicted using ICP spectral data matched the measured values (Fig. 3). The *R*^2^ values for the exchangeable Ca, Mg, K, and Na were 0.995, 0.983, 0.993, and 0.946, respectively. The exchangeable cations were predicted to be similar to those obtained using the official analytical method (i.e., 1 M NH_4_OAc extraction followed by ICP determination). The only difference between the observed and predicted values was the wavelength selection. The actual values were determined using a specific default wavelength set by the ICP management software, whereas our predictions were based on the wavelength monitored at 2574 pixels.

According to Kuang et al.^21^, the most difficult properties to predict using NIR spectroscopy are K and Na. Exchangeable Ca and Mg were relatively difficult to predict, although they were easier to predict than monovalent cations. The *R*^2^ values of NIR predictions for the cations ranged from 0.07 to 0.95 for Ca, from 0.11 to 0.85 for K, from 0.53 to 0.91 for Mg, and from 0.09 to 0.68 for Na^21^. Therefore, these predictions were more accurate than those obtained using NIR. Janik et al.^22^ reported that MIR-based predictions present challenges in determining exchangeable Na and K; however, they can also predict exchangeable Ca and Mg. The *R*^2^ values for Na, K, Ca, and Mg are 0.33, 0.33, 0.89, and 0.76, respectively.

#### Available P and exchangeable Al

**Fig. 4:**
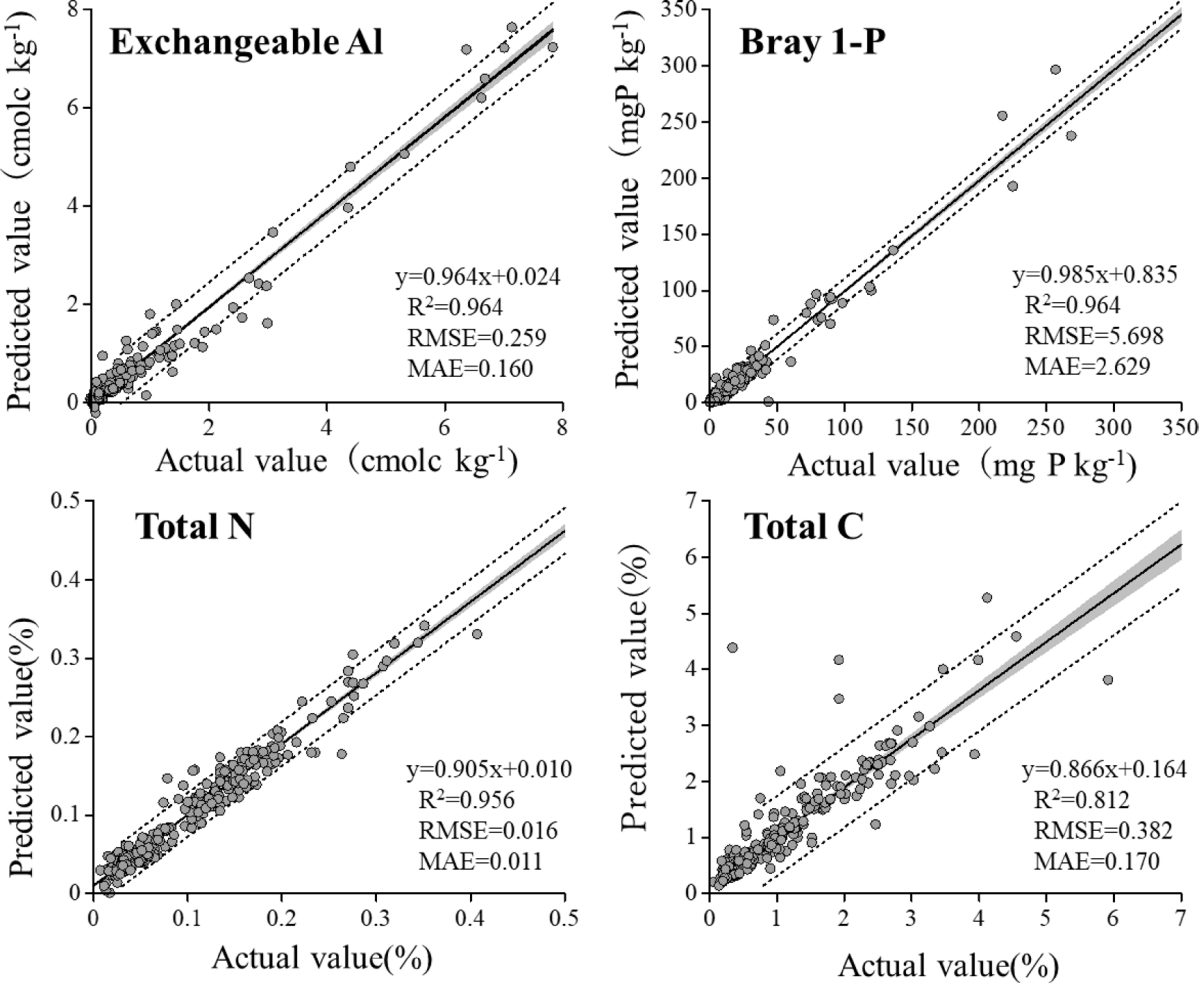
Scatter plots of laboratory-measured values versus ICP spectrum base predicted values of exchangeable Al, Bray1-P, total N amd total C. Grey zones indicate 95% confidence interval, dotted lines indicate 95% prediction interval.

The extract solutions determined using the official method differed from those obtained using the 1 M NH_4_OAc (1 M KCl for exchangeable Al and 0.1 M HCl + 0.03 M ammonium fluoride for Bray1-P) extraction methods. Furthermore, 1 M NH_4_OAc is considered inappropriate for determining exchangeable Al and available P. Nevertheless, we observed that exchangeable Al and Bray-1 P could be predicted accurately using ICP spectrum data (Fig. 4, Ex. Al, *R*^2^ = 0.964; Bray1-P, *R*^2^ = 0.964). Predicting the available P via VIS–NIR is challenging because the low dipole moment between P and oxygen (O) inhibits the direct detection of orthophosphate^11^; therefore, there is no spectral response^23^. However, Abdi et al.^24^ suggested that P can be quantified from VIS–NIR data based on its relationships with a wide range of properties. Few studies have attempted to predict the exchangeable Al content using IR. Janik et al.^22^ reported that MIR could predict exchangeable Al with an *R*^2^ value of 0.64. Therefore, the predictions made using the ICP spectrum were much more accurate than those in previous studies.

#### Total N and C

Figure 4 shows the prediction accuracies for total N and C. The parameters are considered highly difficult to predict using the ICP spectrum because they are unrelated to specific wavelengths for direct determination. In addition, we used an NH_4_OAc solution containing N and C, which could easily mask the peaks of the extracted N and C. Contrary to our expectations, our prediction of total N achieved a high *R*^2^ value (0.956), with an RMSE of 0.016. Total N predictions using MIR spectra have achieved high accuracies, with a median *R*^2^ value of 0.90^11^. The accuracy of our prediction is comparable to these results.

In contrast, our predictions for total C were relatively inaccurate compared to the other parameters: the *R*^2^ value was 0.812, and the RMSE was 0.382. The C content was predicted using the MIR spectrum. Seybold et al.^7^ accurately predicted organic and total C using MIR; they obtained *R*^2^ values of 0.97 and 0.98, respectively. A review paper also reported *R*^2^ values for organic and total C (0.93 in each case)^11^. These accurate predictions are based on significant spectral signatures that occur in the MIR region^25^.

#### Soil pH and EC

**Fig. 5:**
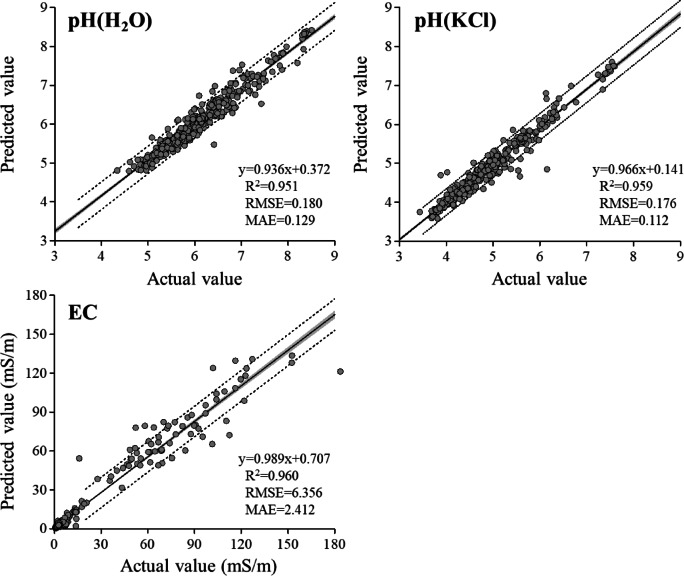
Scatter plots of laboratory-measured values versus ICP spectrum base predicted values of pH (H_2_O), pH (KCl), EC. Grey zones indicate 95% confidence interval, dotted lines indicate 95% prediction interval.

Soil pH is one of the most important parameters affecting nutrient availability, soil biological flora^26^, and other factors. Soil pH is generally determined using the electrode method; thus, it is impossible to determine soil pH using an elemental analyzer such as ICP. However, our predictions yielded very high accuracies for pH values in both water and 1 M KCl (Fig. 5). The *R*^2^ values for the soil pH extracted with H_2_O and 1 M KCl were 0.951 and 0.959, with RMSEs of 0.180 and 0.176, respectively. Seybold et al.^7^ suggested that soil pH predictions made using MIR spectroscopy were inadequate because of the relatively low *R*^2^ values they obtained (0.53), particularly for pH (H_2_O), whereas the prediction of soil pH (CaCl_2_) was more accurate (*R*^2^ = 0.72). Similarly, Janik et al.^21^ obtained an *R*^2^ value for a pH (H_2_O) prediction of 0.56, while that for pH (CaCl_2_) was 0.67. Notably, our method can predict both water- and salt-solution-extracted pH values with high accuracy.

Although the soil EC cannot provide specific ion concentrations, it provides a rough estimate of the available nutrients in a given soil sample. The ICP spectrum-based predictions were highly accurate (*R*^2^ = 0.960, RMSE = 6.356; Fig. 5). The *R*^2^ value for our soil EC prediction was much higher than those of MIR-based predictions, which have a median *R*^2^ value of 0.26^11^.

#### Sand and clay fractions

Particle size distribution (PSD) is a key factor used to evaluate soil fertility because it can be attributed to soil chemical and physical reactions that reflect soil water dynamics. The classical method of analyzing soil particle size distribution is time-consuming, and it is difficult to confirm values that reflect highly skilled procedures, including sample dispersion and pipetting processes. The results of a previous study using MIR prediction suggested that it is possible to predict sand and clay fractions using MIR-based prediction, with median *R*^2^ values of 0.83 and 0.80 calculated from 14 and 11 citations, respectively^11^. In a recent study, novel approaches using the cubist algorithm obtained relatively high accuracies, with *R*^*2*^ values of 0.884 for sand and 0.94 for clay^27^. Although a higher carbonate content (> 5%) in the soil prevents accurate MIR-based prediction in clay fractions^7^, it should be noted that MIR-based predictions can estimate the clay fraction content^28^.

**Fig. 6:**
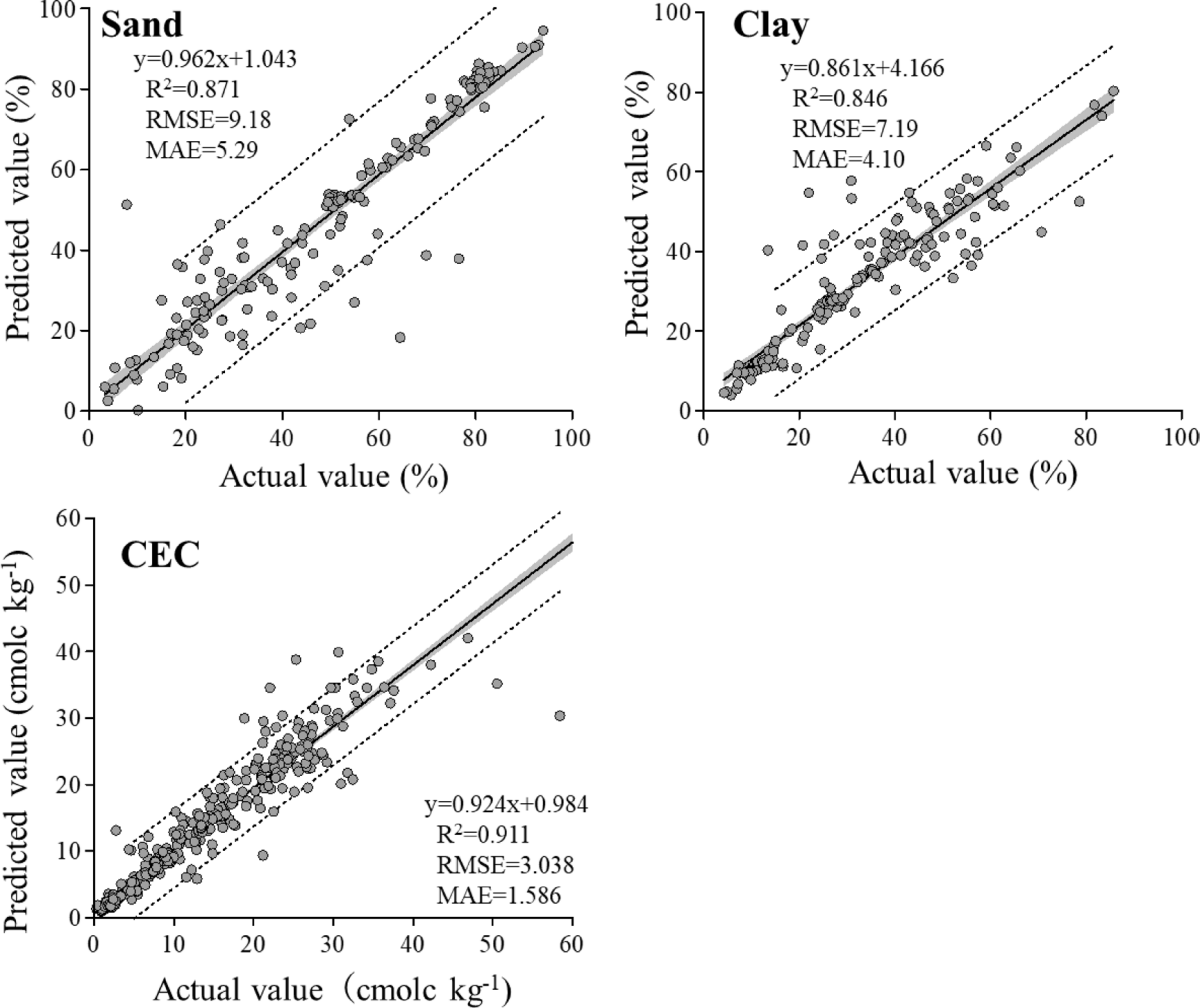
Scatter plots of laboratory-measured values versus ICP spectrum base predicted values of sand fraction, clay fraction, and CEC. Grey zones indicate 95% confidence interval, dotted lines indicate 95% prediction interval.

Figure 6 shows the prediction accuracy obtained using the ICP-based prediction. Our predictions had *R*^2^ values of 0.871 and 0.846 and RMSE values of 9.18 and 7.19 for sand and clay contents, respectively. Although the *R*^2^ values for both fractions were similar to the results obtained from the MIR-based predictions, they were insufficient for soil classification such as soil taxonomy, which requires the detection of a 3% difference^29^. However, the collection of more samples would improve the prediction accuracy.

#### CEC

The relationship between the predicted and observed CEC values is shown in Fig. 6. Our ICP spectrum-based prediction is relatively accurate (*R*^2^ = 0.911, RMSE = 3.038). The CEC is generally related to the amount and type of clay minerals and organic matter in the soil. Although our predictions for total C (Fig. 5) and clay content (Fig. 6) were relatively inaccurate, other elemental parameters, such as exchangeable bases and exchangeable Al, were accurately predicted. The sum of these exchangeable elements is called the effective CEC (ECEC), which is widely used for CEC evaluation, particularly in acidic soils. We believe that the reason for the high accuracy of our method in predicting CEC was the accurate prediction of exchangeable cations and Al, reflecting the existence of a specific wavelength in the ICP determination.

Our prediction accuracy was approximately the same as that of IR-based predictions; studies using MIR-based predictions obtained *R*^2^ values of 0.88^21^ and 0.90^7^. CEC predictions made using the IR spectrum were likely based on the soil surface charges in soil organic matter and clay minerals, whereas our predictions made using the ICP spectrum were probably based on the amount of exchangeable bases.

### Evaluation of the proposed method as a tool for soil diagnosis

The predicted soil properties were evaluated using several guidelines for soil diagnosis. Malley et al.^18^ drafted guidelines using *R*^2^, RPD, and RER. They classified the values of the indices as “Excellent” with values of *R*^2^ > 0.95, RPD < 4, and RER < 20; “Successful” with values of *R*^2^ = 0.90–0.95, RPD = 3–4, and RER = 15–20; “Moderately successful” with values of *R*^2^ = 0.80–0.90, RPD = 2.25–3, and RER = 10–15; “Moderately successful” with values of *R*^2^ = 0.70–0.80, RPD = 1.75–2.25, and RER = 8–0; and “Screening” with values of *R*^2^ < 0.70, RPD < 1.75, and RER < 8. In addition, according to Chang et al.^20^, indices are “Successful” with values of *R*^2^ > 0.8 and RPD > 2.0, “Possibly” with values of *R*^2^ = 0.5–0.8, and RPD = 1.4–2.0, and “Not useful” with values of *R*^2^ < 0.5 and RPD < 1.4.

The results of our soil diagnosis predictions based on Malley’s index are presented in Table 2. Our predictions were all classified as “Excellent” or “Successful,” except for total C, clay content, and sand content (Table 2). As discussed above, it was difficult to predict the total C content because we used an ammonium acetate solution containing C, which masked the C peaks in the ICP spectrum. However, our prediction procedure was likely predictable; according to Chang’s index, our soil diagnosis predictions were “successful” for all parameters. We expect that increasing the sample size will improve the prediction accuracy, including total C. The relatively low accuracy of the prediction of particle size distributions may have been caused by variance in the measured values. As discussed above, it is difficult to determine particle size distributions because the procedures involved are complex. In addition, this is possibly due to the smaller number of measured samples compared to the other parameters.

**Table 2:** Classification of prediction accuracy for soil diagnosis according to previous guidelines.

	pH (H_2_O)	pH (KCl)	EC	Bray1-P	Ex. Al	Ex. Ca	Ex. Mg	Ex. K	Ex. Na	CEC	Total N	Total C	Clay	Sand
			mS m^−1^	mg kg^-1^	cmolc kg^−1^						%			
R^2^	0.951	0.959	0.960	0.963	0.964	0.995	0.983	0.993	0.946	0.911	0.956	0.812	0.844	0.870
RPD	4.51	4.91	4.92	5.17	5.24	12.73	5.68	5.13	3.76	5.10	4.62	2.27	2.54	2.68
RER	23.1	23.6	28.8	47.0	30.2	90.3	56.2	86.2	28.9	19.1	25.1	15.3	11.3	9.9
Accuracy evaluation for soil diagnosis														
Malley’s index	A	A	A	A	A	A	A	A	B	B	A	C	C	D
Chang’s index	A	A	A	A	A	A	A	A	A	A	A	A	A	A

Malley et al. (2004) A: Excellent, B: Successful, C: Moderately Successful, D: Moderately Useful, E: Screening.

Chang et al. (2001) A: Successful, B: Possibility, C: Not Useful.

## Conclusion

We predicted the soil physicochemical parameters using the ICP spectrum of 1 M NH_4_OAc extracts through deep learning. Our results revealed a significant possibility of predicting soil diagnosis using ICP spectra, and the predicted parameters generally matched the observed parameters. Our soil diagnosis predictions were generally successful according to previous guidelines. These results should be considered the first step, which uses a simple learning procedure with a limited sample size. Increasing the sample size could further improve the prediction accuracy. Further studies using a complete dataset in which ICP is collected using a charge-coupled device detection unit (almost 1 megapixel) and in which learning is conducted using a convolutional neural network (CNN) may provide a more accurate prediction. Therefore, comparative studies with alternative architectures should be conducted for future work. Moreover, in this study, we used NH_4_OAc as the extractant; however, we intend to investigate the selection of more suitable extraction solutions in future work, which could potentially lead to improved prediction accuracy.

We evaluated our prediction accuracies by comparing them with IR spectral data methods such as MIR. These techniques can be used for real-time, non-destructive, and non-contact soil diagnosis. In the near future, cutting-edge technologies will be used globally in precision agriculture. However, to date, these methods have not successfully predicted exchangeable bases. In addition, we believe that wet extraction soil diagnosis will continue to be a major technology, and that our proposed method can be another approach.

Our prediction procedure can make soil diagnosis more affordable than standard methods by reducing various costs, such as labor, time, and chemical costs. This could enhance the soil diagnostic activity, particularly for farmers in developing countries. In addition, ICP spectrum-based predictions can facilitate laboratory-based soil chemical analyses because an approximate concentration range can be obtained in advance. We believe that this approach can be used to predict soil health based on various parameters, including physicochemical properties and soil biodiversity, and to predict soil pollution, including heavy metals, microplastics, and PFAS (Per- and Polyfluoroalkyl Substances). However, ICP remains unrealistic in some developing countries because it requires a high-frequency power supply and high-purity Ar gas. Further studies using applicable and affordable plasma spectrometry techniques are required.

## Supplementary Information

Below is the link to the electronic supplementary material.

Supplementary Material 1

Supplementary Material 2

## Data Availability

Data is provided within the manuscript or supplementary information files.
