# Supplementary material for: Deep learning using inductively coupled plasma spectroscopy spectra accurately predicts various soil physicochemical properties for soil diagnosis
Source: Sci Rep. 2025 Nov 20;15:37753. doi: 10.1038/s41598-025-24274-3 (PMC12634687; doi:10.1038/s41598-025-24274-3)
Supplement: Supplementary file 1 — Supplementary Material 1 [file 41598_2025_24274_MOESM1_ESM.pdf]

Suppl. table 1 Sample numbers from each country subjected to soil parameter analysis

Country	pH (H_2O)	pH (KCl)	EC	Bray I-P	Total -N	Total -C	Cation	CEC	Ex-Al	Clay	Sand
Burkina Faso	1073	1073	1073	1074	947	947	1074	1074	516	348	342
Laos	334	334	334	334	334	334	334	334	334	334	334
Japan	298	298	298	298	298	298	298	298	0	48	48
Mozambique	115	115	115	115	115	115	115	115	71	115	115
Palau	59	59	59	59	59	59	59	59	59	0	0
Madagascar	49	49	49	49	49	49	49	49	49	0	0
Philippines	13	13	13	13	13	13	13	13	13	13	13
Total	1941	1941	1941	1942	1814	1814	1942	1942	1042	858	852
