# Supplementary material for: Deep learning using inductively coupled plasma spectroscopy spectra accurately predicts various soil physicochemical properties for soil diagnosis
Source: Sci Rep. 2025 Nov 20;15:37753. doi: 10.1038/s41598-025-24274-3 (PMC12634687; doi:10.1038/s41598-025-24274-3)
Supplement: Supplementary file 2 — Supplementary Material 2 [file 41598_2025_24274_MOESM2_ESM.pdf]

Suppl. table 2 Highest and lowest correlation coefficients between observed soil parameters and 234 logarithmically transformed wavelength intensities (pixel point 0)

	pH(H_2O)	pHKCl	EC	Bray1P	Bray2P	T-N	T-C	Ca	K	Mg	Na	Al	CEC	Clay	Sand
Max Correlation	0.482	0.358	0.456	0.442	0.487	0.370	0.339	0.495	0.245	0.313	0.383	0.240	0.471	0.366	0.266
Wavelength	337.280	337.280	330.232	177.499	766.490	396.847	396.847	396.847	323.261	330.232	330.232	269.206	267.876	291.139	274.716
Default Element	Ti	Ti	Na	P	K	Ca	Ca	Ca	Li	Na	Na	Ru	Ru	Lu	Th
Min Correlation	-0.186	-0.348	-0.190	-0.009	-0.144	-0.058	-0.152	-0.225	-0.260	-0.210	-0.276	-0.352	-0.203	-0.229	-0.437
Wavelength	242.220	242.220	231.604	233.527	233.527	379.478	383.231	257.610	383.231	383.231	383.231	268.517	383.231	274.716	291.139
Default Element	Y	Y	Ni	Ba	Ba	La	Mg	Mn	Mg	Mg	Mg	Ta	Mg	Th	Lu
